# LdsConv: Learned Depthwise Separable Convolutions by Group Pruning

**DOI:** 10.3390/s20154349

**Published:** 2020-08-04

**Authors:** Wenxiang Lin, Yan Ding, Hua-Liang Wei, Xinglin Pan, Yutong Zhang

**Affiliations:** 1 Key Laboratory of Dynamics and Control of Flight Vehicle, Ministry of Education, School of Aerospace Engineering, Beijing Institute of Technology, Beijing 100081, China; wenxianglineut@163.com (W.L.); zhang1123034978@163.com (Y.Z.); 2 Department of Automatic Control and Systems Engineering, University of Sheffield, Sheffield S1 3JD, UK; w.hualiang@sheffield.ac.uk; 3 Department of SMILE Lab, School of Computer Science and Engineering, University of Electronic Science and Technology of China, Chengdu 610031, China; kp600168@gmail.com

**Keywords:** convolutional neural network, convolutional filter, classification

## Abstract

Standard convolutional filters usually capture unnecessary overlap of features resulting in a waste of computational cost. In this paper, we aim to solve this problem by proposing a novel Learned Depthwise Separable Convolution (LdsConv) operation that is smart but has a strong capacity for learning. It integrates the pruning technique into the design of convolutional filters, formulated as a generic convolutional unit that can be used as a direct replacement of convolutions without any adjustments of the architecture. To show the effectiveness of the proposed method, experiments are carried out using the state-of-the-art convolutional neural networks (CNNs), including ResNet, DenseNet, SE-ResNet and MobileNet, respectively. The results show that by simply replacing the original convolution with LdsConv in these CNNs, it can achieve a significantly improved accuracy while reducing computational cost. For the case of ResNet50, the FLOPs can be reduced by 40.9%, meanwhile the accuracy on the associated ImageNet increases.

## 1. Introduction

Convolutional neural networks (CNNs) have shown remarkable achievements in various vision tasks [1,2,3,4,5,6,7,8]. Most of the achievements benefit from the innovative design of network architectures [9,10,11,12,13,14], with applications in a variety of areas including phishing detection (see, e.g., [15]). Recent designs usually use the convolutional filter as the basic unit and achieve good training results through special network architectures. However, the manual design of the network architecture has been gradually replaced by architecture searching [16,17,18,19,20,21,22] with the rapid development of the computation ability of the hardware. Compared with architecture searching, which often requires strong computing power and expensive time cost, the model compression method and other new convolutional filter design techniques [23,24,25] provide an economic choice to improve the efficiency of CNNs.

At present, the commonly used convolutions are Groupwise Convolution [2], Depthwise Convolution [26] and Pointwise Convolution [27]. Pointwise Convolution is able to adjust the dimension of the channels or feature maps. It is widely used in the design of architectures. Groupwise Convolution can reduce the connection density and computation cost of convolutional filters, while Depthwise Convolution is the extreme version of Groupwise Convolution which sets the number of groups to be the same as the number of input channels. However, if we simply replace the standard convolution with Depthwise or Groupwise Convolution without special adjustment of the architecture, the resulting model may not work well. Therefore, some new convolutional filters have been proposed recently. HetConv [23] proposes the heterogeneous kernel-based convolution. OctConv [24] designs a convolutional filter that can extract multi-scale information from features. These convolutional filters have the ability to improve the performance of model by simply replacing the standard convolutions without any adjustment of the baseline. The present study proposes a similar but different plug and play convolutional unit. Our proposed LdsConv pays more attention on the learning ability of the model and aims to transform a standard convolutional filter into a learned depthwise separable convolutional filter.

Model compression is considered as another reliable and economic method to improve the efficiency of the convolutional neural network, which can be roughly divided into three categories: (a) Connection pruning [28,29]; (b) Filter pruning [30,31,32,33,34,35,36]; and (c) Quantization [28,37,38,39]. These methods can effectively reduce the computation of the convolutional neural network, but this is always achieved at the price of sacrificing the accuracy. Sometimes, special hardware support is also required for compression methods.

Instead of directly pruning the whole model, we choose to integrate the pruning technique into the design of convolutional filters. In this way, the model can automatically learn to know which input features are most valuable for each single output, so that it enables to extract better features with fewer filters. To achieve this objective, we design a new type of convolutional filter—Learned Depthwise Separable Convolution (LdsConv), which can be directly plugged into existing standard architecture to reduce floating point of operations (FLOPs) and meanwhile improve the accuracy.

To integrate the pruning methods, we develop the two-stage training framework to divide the training task into picking and combining. In the first stage, the LdsConv picks out the most valuable input features and applies more filters to them by pruning technique. In the second stage, the additional pointwise convolution combines the output of the first stage and produces the output features. The idea of division of labour and progressive working has been reflected in computer vision. For example, the two-stages detection framework [40] divides the task into region proposed stage and classification as well as location stage. Cascade RCNN [41] further refines the second stage into three parts and each part is based on the front one. Similarly, we adopt this idea in the convolutional operation and thus divide the training task into picking up useful filters and mixing up the results of picking up. The relationship between two stages is progressive and inseparable. The two-stage training process simplifies the training task for each stage and finally improves the efficiency of the model.

Our experiments show that by replacing the standard/depthwise convolution with the LdsConv in CNNs, it can improve the accuracy and reduce computational costs in the following models: ResNet [1], DenseNet [42], MobileNet [9], and SE-ResNet [43].

Our main contributions are three-fold:

1. We integrate the weight pruning method into the depthwise separable convolutional filter and develop the two-stage training framework.
2. We design an efficient convolution filter named Learned Depthwise Separable Convolution, which can be directly inserted into the existing CNNs. It can not only reduce and computational cost, but also improve the accuracy of the model.
3. We validate the effectiveness of the proposed LdsConv through extensive ablation studies. To facilitate further studies, our source code, as well as experiment results, will be available at https://github.com/Eutenacity/LdsConv.

## 2. Related Work

### 2.1. High Efficiency Convolutional Filter

Ever since the pioneering work on Alexnet [2] and VGG [3], researchers have studied how to improve the efficiency of CNNs from various perspectives. However, much less work has been devoted to developing innovative convolutional filters. Among those proposed convolutional filters, the most popular ones are Groupwise Convolution [2], Depthwise Convolution [26] and Pointwise Convolution [27]. They are widely used in the design of efficient CNNs. ResNet [1,44] uses Pointwise Convolution to build bottleneck layers that allow the network to go deeper without increasing too many parameters. For example, ResNeXt [45] and ShuffleNet [12] use Groupwise Convolution to reduce redundancy in internal connections. Xception [10] and Mobilenet [9] use Depthwise Convolution to further reduce the connection density. SENet [43] and CBAM [46] design a module that can automatically weigh the output of convolutional filters at the cost of a small number of parameters. Hetconv [23] uses convolutional filters with heterogeneous kernels to replace the standard convolutional filters. OctConv [24] reduces the spatial redundancy in CNNs by designing special convolutional filters with multi-scale input features. The Multi-Kernel Depthwise Convolution proposed in [47] can better extract information with multiple kernel sizes and effectively utilize the computational efficiency of Depthwise Convolution. The fully learnable group convolution (FLGC) proposed in [48] can be integrated into a deep neural network and automatically learn the group structure in the training stage in a fully end-to-end manner; its can achieve high computational efficiency. In [49], a new dynamic grouping convolution (DGConv) was proposed, which is able to learn the number of groups in an end-to-end manner; it has been proven to have several advantages. The training-free method, called network decoupling (ND), proposed in [50] is interesting; it achieves high computational efficiency and accuracy performance via pre-trained CNN models which are transferred to the MobileNet-like depthwise separable convolution structure. Compared to these methods, the proposed LdsConv chooses to incorporate weight pruning technique into the design of convolutional filters and further develops the two-stage training framework to simplify the training task for each stage.

### 2.2. Model Compression

Model compression is another popular method to improve the efficiency of the convolutional neural network. Refs. [28,29] remove redundancy in the model by pruning connection. Refs. [28,37,38,39] compress the calculation amount of the model via quantization. Refs. [30,31,32,33,34,35,36] prune filters that have a minimal contribution in the model. After removing these filters, the model is usually fine-tuned to maintain its performance. Among these methods, filter pruning methods generally do not require special hardware and software, but they need a pre-trained model which may use a computationally expensive training to obtain.

The proposed LdsConv inserts the weight pruning process into the training. Therefore, the LdsConv embedded model is able to be trained from scratch without a pre-trained model. Different from [51] which only integrates the pruning and fine-tuning process with training, LdsConv further develops the two-stage training framework dividing the training task into picking and combing. Moreover, LdsConv conducts the group pruning by replacing the original convolution with the groupwise convolution before training and use an additional balanced loss function to make the pruning procedure more smooth. Additionally, LdsConv adds an additional pointwise convolution at the end of the pruning, to integrate the pruning results and build a regular depthwise separable convolution, allowing for efficient computation in practice at test time.

## 3. Method

In this section, we first introduce Depthwise Separable Convolution and LdsConv. Then we describe the details about the utilization of LdsConv. We also discuss implementation details and show how to replace Depthwise Separable Convolution with LdsConv.

### 3.1. Depthwise Separable Convolution

Consider a standard convolution that takes an $R\times D_{h}\times D_{w}$ feature as an input and produces an $O\times D_{h}\times D_{w}$ feature as an output, where *R*, *O*, $D_{h}$ and $D_{w}$ denote the numbers of input channels, output channels, and the height and the width of the feature. Usually a standard convolution applies *R* filters to every input channel for each output. Thus, a standard convolution has the weight matrix with the size of R×O×H×W where *H* and *W* denote the height and the width of the filter. To reduce the computational cost, the depthwise separable convolution splits the standard convolution into two: a depthwise convolution for filtering, that only applies a single filter to the corresponding input channel for the output one, and a pointwise convolution for combing the outputs of the depthwise convolution and producing final output channels. The depthwise convolution is parameterized by the kernel of the size R×1×H×W and the pointwise convolution is of the size R×O×1×1.

### 3.2. Learned Depthwise Separable Convolution

**Figure 1:**
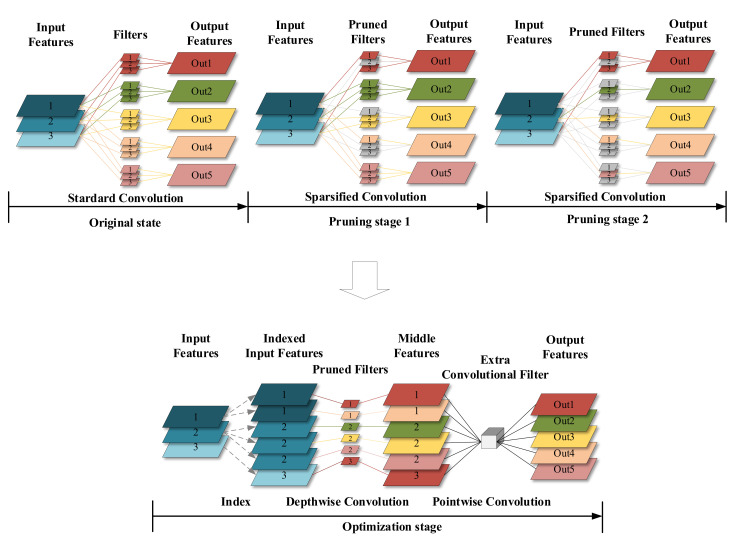
The illustration of the LdsConv with a input channel of R=3, an output channel of O=5, a group cardinality of $N_{O}=5$, a group number of G=1, a pruning factor of k=2 and a stage factor of s=2. At the end of picking stages, we remove filters with the number of $(N_{O}-k)R$. After the picking stages, an additional 1 × 1 standard convolution is added into the convolutional module to form a standard depthwise separable convolution.

Considering the strength of the depthwise separable convolution, it is highly desirable to design a more complex architecture to enhance the capability of the convolution so that the neural network can decide on which feature should be applied. In doing so, we need a novel convolution architecture, named Learned Depthwise Convolution (LdsConv). As shown in Figure 1, the training process is divided into picking stages and the combining stage. Moreover, the training task is also divided into picking and combining. In picking stages, we focus on removing little influence filters repeatedly to pick out valuable input features. In the combining stage, similarly to Depthwise Separable Convolution, an additional 1 × 1 convolution is applied to combine features.

#### 3.2.1. Group Pruning

Initially, we adopt a group convolution which divides a standard convolution of size R×O×H×W into G groups of 4D tensors $F^{g}$ with the size of $N_{R}\times N_{O}\times H\times W$ to initialize our architecture. For convenience of description, define $N_{R}=\frac{R}{G}$ and $N_{O}=\frac{O}{G}$. Given the fact that the size of convolution layers is widely different which needs different G for the division operation, in the experiment we set a unify hyper-parameter $N_{O}$, named group cardinality, to represent our model and analyze its influence on the accuracy. Group pruning aims to relieve the effect of the pruning to the accuracy by making pruning results more uniform.

#### 3.2.2. Pruning Criterion

During the training process, we gradually screen out less important filters for each group. The importance of the filters is evaluated by the $L_{1}$-norm of its weight $F^{g_{ij}}$ that corresponds to the weight of the i-th input for the j-th output within group g. In other words, we remove filters with the $L_{1}$-norm.

#### 3.2.3. Pruning Factor

It is important to consider and determine how many filters should be removed before the combining stage. Formally, we set a hyper-parameter *k* with a range from 1 to 4 to represent that the number of remaining filters is k×R. In Section 4, discussions and analysis on how to choose *k* is presented, which both has a good balance of parameter and accuracy and fits all around dataset and network scale.

#### 3.2.4. Stage Factor

In contrast to methods that prune weights in pre-trained models, our weight pruning process is plugged into the training procedure. Thus, we define the stage factor to determine the times of pruning. For a group filter weight $F^{g}$ with size of $N_{R}\times N_{O}\times H\times W$, the number of filters that need to be pruned can be calculated by the equation $N_{d}=N_{R}N_{O}-kN_{R}$. Thus, the total number of pruned filters is $GN_{d}=RN_{O}-kR$. Then, at the end of each picking stage, we prune $GN_{d}/s$ filters.

#### 3.2.5. Balance Loss Function

To reduce the negative impact on the accuracy induced by pruning, we deliberately set the number of remaining filters of each input feature to be even avoid the case that most of remained filters extract information from only a small number of input features. As we know, it is hard to optimize the number of filters as they are non-differentiable. We thus define the coefficient of *M* to ensure that filters belong to input features with a bigger number of possible remained filters would be penalized more strongly.

In each training iteration in picking stages, we first find the filters that have the highest probability to remain. Then, we check their input features to get the number of probably remaining filters of each input feature. Finally, we restrain these filters belong to input features having a big number of probably remaining filters. To this end, we use the following regularizer for a group filter weight $F^{g}$ during training:

(1) $$L_{bal}=\sum_{j=1}^{N_{O}}\sum_{i=1}^{N_{R}}M_{i}(\sum_{l=1}^{HW}\left|w_{l,i,j}\right|{)}^{2}$$

where $M_{i}$ denotes the coefficient for filters belong to the i-th input feature and $w_{l,i,j}$ denotes every parameter in $F^{g_{ij}}$. By adjusting the coefficient of $M_{i}$, the input feature having higher number of probably remaining filters will force its filters to be penalized more strongly. The equation for $M_{i}$ is defined as:

(2) $$M_{i}=max\left(e^{(N_{i}^{R}-\lambda k)/\gamma }-1,0\right)$$

where $N_{i}^{R}$ denotes the number of probably remaining filters belonging to the i-th input feature. We introduce a parameter λ to define the threshold over which the filter belonging to the i-th input feature will receive the penalty since the average value of $N_{i}^{R}$ is *k*. Furthermore, γ is set to adjust the penalty level. In this paper, we set λ=1.5 and γ=10 in all experiments empirically.

#### 3.2.6. Additional Pointwise Convolution

**Figure 2:**
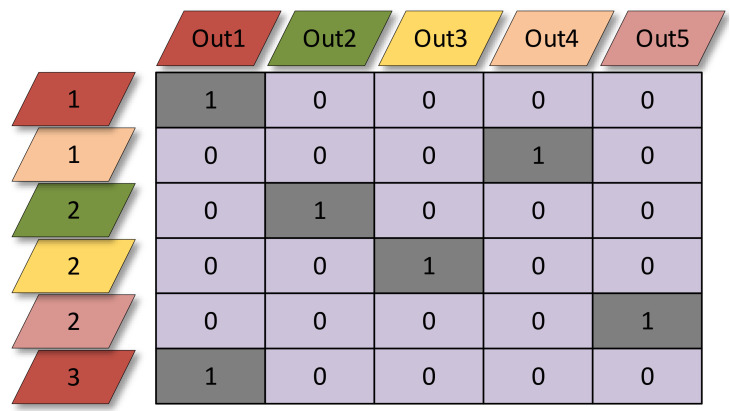
Initial value assignment of the example shown in Figure 1. The left set of parallelograms represents the middle features. The numbers in these parallelograms mean the index in the input features. The upper set of parallelograms represents the output features. The same color between the left parallelogram and the top one means that they are matched in picking stages. The value in the matrix means the initial value of the additional convolution.

At the end of picking stages, we convert the sparsified model into a network with regular modules that can be efficiently deployed on devices without special hardware and software support. For this reason, we add additional pointwise convolutions to each LdsConv to build Depthwise Separable Convolution (see Figure 1). This operation also highly broadens the expression ability of LdsConv filters and lead the training task to combining the output of picking stages and producing the final output features. The weight of the additional pointwise convolution has the size of kR×O×1×1 related to the number of input channel *R* and output channel *O* of the original convolution and the pruning factor *k*. The initial value of the weight is set by the index information of the remaining filters. Figure 2 shows the initial value of the example in Figure 1. We set the value of the position in the weight matrix to 1 only when the middle feature extract by the remaining filter matches the output feature. The color in Figure 1 represents this matching relationship. This kind of initial value can narrow the negative effect of the newly additional pointwise convolution added in the training process.

#### 3.2.7. Learning Rate

**Figure 3:**
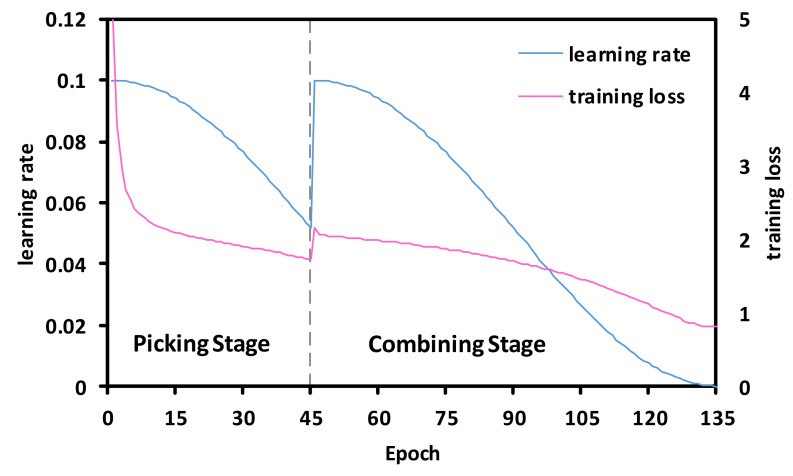
The cosine shape learning rate and a typical training loss curve trained on ImageNet. The vertical gray bar in the figure marks the end of picking stage and the begin of combing stage.

We adopt the cosine shape learning rate schedule during training, which smoothly changes the learning rate, and usually improves the accuracy [18,52,53]. Figure 3 demonstrates the learning rate as a function of training epoch, and the corresponding training loss of a ResNet50 using LdsConv filters on the ImageNet dataset [54]. Before we enter the combining stage, we add additional pointwise convolution and reset the learning rate to reduce the negative effect of the learning rate to the newly added weights. Thus, the abrupt increase occurs in the loss at epoch 45. However, the plot shows that the loss gradually recovers from this accident.

### 3.3. The Implementation of LdsConv

In addition to the use of LdsConv, we briefly describe how to replace standard convolutional filters and depthwise separable convolutional filters with LdsConv filters.

#### 3.3.1. Standard Convolution

**Figure 4:**
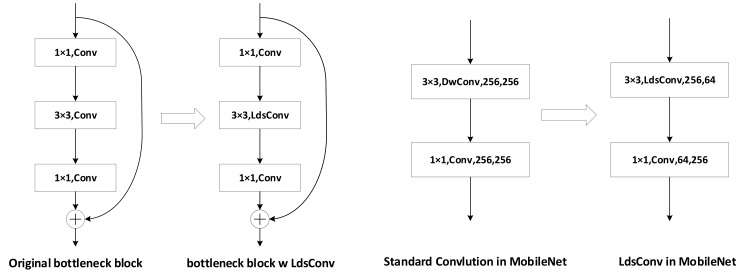
The replacement of original convolutional filters with LdsConv. Left: The replacement in ResNet. We directly replace the 3 × 3 convolution with our proposed LdsConv in the bottleneck block. Right: The replacement in MobileNet. We replace the original 3 × 3 convolution and reduce the number of output channel of the LdsConv and input channel of the sequent 1 × 1 convolution.

When we try to replace a standard convolution with our proposed LdsConv, the most important hyper-parameter is the group cardinality $N_{O}$. In general, we suggest setting $N_{O}$ to the value from 8 to 32. But if the number of the channels of the original convolution is too small to divide, we need to set $N_{O}$ to the same value as the number of output channels ensuring the group to be 1. For other hyper-parameters, we can simply use the recommended value given by Section 4. In addition to the fact that we replace the standard convolution with the group one first, a 1 × 1 convolution should exist to mix all channels information after the group convolution. In Figure 4, we demonstrate the replacement in the ResNet.

#### 3.3.2. Depthwise Separable Convolution

In general, a pointwise convolution exists in each depthwise separable convolution. So, we do not need to worry about the problem mentioned above. In other words, we can simply replace the depthwise convolution with our proposed LdsConv. However, parameters and FLOPs may increase if we do not make any adjustments. Therefore, we suggest adding an additional convolution before or after the LdsConv to reduce the number of input or output channels of the LdsConv. The right part of Figure 4 shows our implementation of LdsConv filters in MobileNet.

## 4. Experiment

In this section, we validate the effectiveness and efficiency of the proposed LdsConv. We first present ablation studies for image classification on Cifar [55]. Then, we perform a set of experiments on ImageNet [54] to check the performance of the proposed LdsConv.

### 4.1. Ablation Study on Cifar

We conduct a series of ablation studies to find the best situation to implement LdsConv filters and then check its robustness in different models.

#### 4.1.1. Training Details

We use stochastic gradient descent (SGD) algorithm to train all the models. Specifically, we adopt Nesterov momentum with a momentum weight of 0.9 without dampening, and use a weight decay of $1e^{-4}$. Unless otherwise specified, the size of the training batch is set to be 64 and the number of total training epochs is 300, in which the picking stages take 150 epochs and the combining stage has 150 epochs. For the convenience of network accuracy comparison, we all use the standard cosine learning rate change strategy without reset which starts from 0.1 and gradually reduces to 0. It is worth mentioning that special modification on learning rate dose not affect too much. Therefore, we remove the reset described in Section 3.2.7 for the convenience.

#### 4.1.2. Implement on DenseNet-BC-100

We do experiment with DenseNet-BC-100 architecture having a growth rate of 12 [42] on the CIFAR-100 dataset.When we implement our proposed LdsConv, we simply replace the 3 × 3 convolutional filters in dense blocks with the LdsConv filters. Specifically, we set the group cardinality $N_{O}$ to the same as the number of output channels since the number is too small to divide. Then we start experiments on the effect of pruning factor *k* and stage factor *s* for the LdsConv.

#### 4.1.3. Effect of Stage Factor

The first part of Table 1 compares DenseNet-BC-100 models having LdsConv filters with different stage factors. In particular, we set the pruning factor *k* to 2. The result shows that s=4 seems to be the best value. While reaching the peak at 4, the accuracy drops down for higher stage factors. We attribute this change to the decreasing of gap epochs between pruning which is calculated by the equation $E_{G}=E_{P}/s$ where $E_{P}$ denotes training epochs of picking stages. To expel its effect, we conduct two more experiments with s=6 and s=8 and set $E_{G}$ to be the same value as the one when s=4 in the second part of Table 1. In other words, the picking stages of these two experiments take 225 and 300 epochs, respectively. The result shows that the accuracy can increase a lot without the decreasing of gap epochs $E_{G}$. By taking into account the training time, we suggest to set the stage factor to 4 in the ordinary course of events.

#### 4.1.4. Effect of Pruning Factor

We do experiment with several pruning factors *k*, which vary from 1 to 4. In addition, we set the stage factor *s* to 4 which means all models have the same times of pruning. The results presented in the third part of Table 1 demonstrate that parameters of the model raise while the accuracy rise ups and downs with the increasing of the pruning factor. The risk of overfitting and the decreasing of pruning proportion battle with each other resulting in this change. In particular, it suggests that setting the pruning factor *k* to 2 is a good choice which balances both the accuracy and the number of parameters. We can also reduce the pruning factor *k* to 1 or even integrate the additional pointwise convolution with the sequent convolution to reach a higher reduction to weights.

#### 4.1.5. Effect of Balance Loss Function

To check the effectiveness of our balance loss function, we apply it to the models with varied pruning factors. The fourth part of Table 1 shows that the accuracy is improved by adding the balance loss regularization.

#### 4.1.6. Effect of Group Cardinality

To evaluate the effect of the group cardinality $N_{O}$, we experiment with ResNet50 [1] which is designed to train on ImageNet and thus has large number of channels. We remove the first three downsampling operations and retain only the last two ones since images in cifar have smaller resolution. The fifth part of Table 1 compares ResNet50 models using LdsConv filters with varied group cardinality. Specifically, we set the group cardinality $N_{O}$ to 4,8,16 and 32. The stage factor *s* is set to 4 and the pruning factor *k* is set to 2 for all models. The result shows that the accuracy first rises up and then goes down. When $N_{O}=8$, the model reaches its best accuracy. While reaching the accuracy peak at 8, the accuracy drops down for lower $N_{O}$ indicating over-group can also have negative effects. We own the negative effects to the shrink in expression ability when the convolution is grouped.

#### 4.1.7. Effect of Two-Stage Training Framework

**Figure 5:**
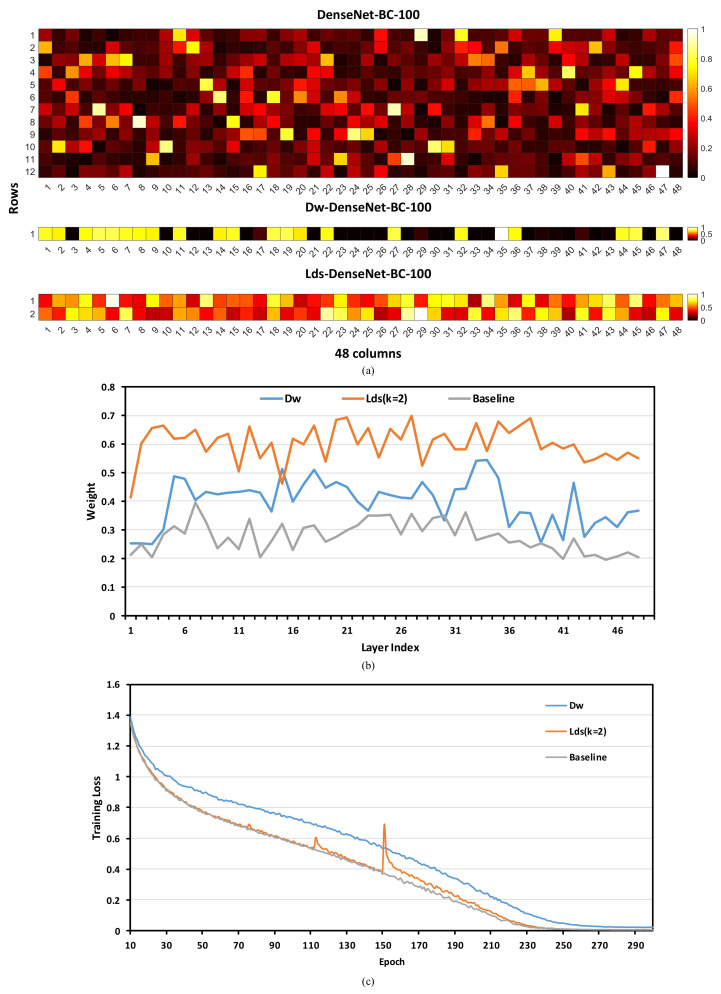
(**a**): Norm of weights of three models in Cifar 100. The block with darker color has less value of norm. The vertical and horizon axis represent the height and width of the weight matrix expect the Lds-DenseNet-BC-100 in which we arrange it in this way for alignment. (**b**) The curve between 48 3 × 3 convolutional layers in dense blocks and the average norm of weights for three models. (**c**) The curve of convergence speed for three models.

To verify the function of each stage, we first explore the norm value of the picking results and then evaluate the effect of the additional convolution. The three panels of Figure 5a illustrates the weights of the last 3 × 3 convolution for orignal DenseNet-BC-100, Dw-DenseNet-BC-100 and Lds-DenseNet-BC-100. We replace the 3 × 3 standard convolutions in dense blocks with depthwise separable convolutions in Dw-DenseNet-BC-100 which can be regarded as the typical one-stage training form of LdsConv. Each block in the figure represents the L1 norm (normalized by the maximum value among all filters) of a 3 × 3 filter. In the top two panels of Figure 5a, the vertical and horizon axis represent the height and width of the weight matrix, respectively. For the third panel, we arrange the weight matrix of Lds-DenseNet-BC-100 in this way for alignment. Figure 5b shows the curve between 48 3 × 3 convolutional layers in dense blocks and the average norm of weights for three models. The results suggest that the picking stage indeed reduces the redundancy in the weight matrix and picks up more valuable filters. We additionally experiment with Dw-DenseNet-BC-100 and Lds-DenseNet-BC-100 (*k* = 2) without additional convolutions (AC) in the final part of Table 1. Without additional convolutions, the combing stage becomes the common optimization one. The accuracy dramatically drops down indicating that the combing stage is indispensable. Furthermore, additional convolutions arrange the sparsified convolutions into standard depthwise separable convolutions improving the computation cost at test time. Besides, Dw-DenseNet-BC-100 shows lower accuracy and non negligible gap in the convergence speed compared with the baseline in Figure 5c. On the contrary, Lds-DenseNet-BC-100 trained with the two-stage training framework owns a better curve of convergence speed which is near to the baseline.

#### 4.1.8. Results on Other Models

To evaluate the effectiveness of the proposed LdsConv with the situation discussed in the above in different networks, we choose currently popular models as the baselines including ResNet [1], DenseNet [42], MobileNet [9], and SE-ResNet [43]. For all experiments, we set the pruning factor *k* to 2, the stage factor *s* to 4 and the balance loss function active. In DenseNet, we set its pruning cardinality $N_{O}$ to the same value as the number of output channels. In other networks, we set the pruning cardinality $N_{O}$ to 8. The experimental results are shown in Table 2. After using our modules to replace the convolutions in the original models, these networks generally achieve the effect of reducing the FLOPs and the number of parameters, meanwhile maintaining or even improving the accuracy. It shows that our method can effectively reduce the redundancy in convolutional filters. It also suggests that the LdsConv can perform well without too many adjustments on hyper-parameters.

### 4.2. Results on ImageNet

In a set of experiments, we test LdsConv filters on the ImageNet dataset.

#### 4.2.1. Training Details

We use the SGD method to train all the models and adopt Nesterov momentum with a momentum weight of 0.9 without dampening using a weight decay of $1e^{-4}$. We use 135 as the total training epochs, in which the picking stage takes 45 epochs, the combining stage involves 90 epochs. The learning rate change strategy is shown in Figure 3. For MobileNet, we choose to simply increase the training epochs rather than adjusting hyper-parameters to the best. Thus, we use 300 as the total training epochs, in which the epoch size of the picking stage and combining stage is set as 100 and 200, respectively. The initial learning rate is 0.045, and its weight decay is $4e^{-5}$.

#### 4.2.2. Model Configurations

In the experiments on ImageNet, we set the balance loss function active, the pruning factor *k* to 2 and the stage factor *s* to 4. Except for DenseNet, we set the group cardinality $N_{O}$ to 8. In DenseNet, we still set its group cardinality to the same value as the number of output channels ensuring the group to be 1.

#### 4.2.3. Comparison on ImageNet

We continue to use ResNet [1], DenseNet [42], MobileNet [9], and SE-ResNet [43] as the baseline for comparison, and the results are shown in Table 3. All results of baselines come from their original papers. In MobileNet, we can slightly reduce parameters and FLOPs, and highly increase the accuracy by 2.3%. For other networks using standard convolution originally, we not only improve the accuracy but also obviously reduce the number of parameters and FLOPs. What’s more, our modules can coexist with SE-modules to further improve the efficiency of the model.

#### 4.2.4. Comparison with Model Compression Methods

**Figure 6:**
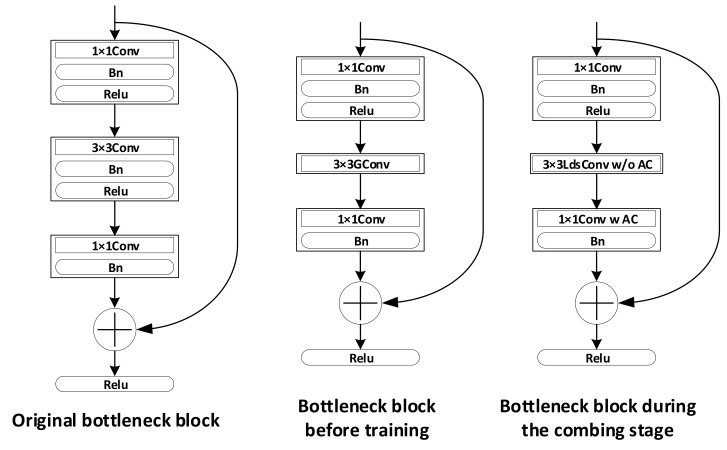
The extreme state of LdsConv in the ResNet. We remove the Bn and Relu layer after the 3 × 3 convolution and combine the additional convolution with the sequent 1 × 1 convolution by the matrix multiply operation. Finally the standard convolution is replaced with only depthwise convolution. The 3 × 3 LdsConv w/o AC means the depthwise part in LdsConv. The sequent 1 × 1 Conv w AC means the combing result of the additional convolution and original sequent 1 × 1 convolution.

To investigate the compressing ability of our proposed LdsConv, we adjust the bottleneck block with LdsConv in the ResNet to a extreme state as shown in Figure 6. To this end, we remove the Bn and Relu layers after the 3 × 3 group convolutional layer before training. When the combination stage begins, we integrate the additional pointwise convolution (AC) with the sequent 1 × 1 convolution by the matrix multiply operation since no non-linear operation exists between them. When the model formally enters the combing stage, we only train one 1 × 1 convolution after every LdsConv. In Table 4, we compare the LdsConv with the existing compression methods including ThiNet [30], NISP [56] and FPGM [57]. We use ResNet50 as the baseline, replace the standard convolution with the LdsConv, and reduce the number of parameters further by setting the pruning factor to 1 and combing the additional pointwise convolution with the sequent 1 × 1 convolution. We also set s=6 and $E_{G}=E_{P}/4$, which lengthens the training epochs, in order to relieve the negative effect of extremely compressing. Compared with these pruning methods, our method, denoted as Lds-ResNet50-extreme, not only improves the accuracy outperforming all other compared methods but also reduces the FLOPs by 40.9%. Furthermore, the real inference speed of Lds-ResNet50-extreme is 42 batches (16 images per batch) per second with the practical evaluation on GPU Nvidia RTX 2080 compared with the 28.9 batches per second on the baseline of ResNet50. We can obtain nearly 1.5× speed up without special hardware support.

### 4.3. Comparison with Similar Works

To further verify the effectiveness of our approach, we do several experiments using three different networks, namely, ND [50], FLGC [48] and GDConv [49] as well as the proposed model. A comparison of the four models is shown in Table 5. These methods perform similarly when they transform a regular convolution into a depthwise/groupwise convolution. To fairly evaluate the performance of each method, we reimplement these methods in ResNet50 since they have different baselines in their original papers. FLGC mainly transforms the 1×1 convolution into groupwise one and thus can reduce the FLOPs a great deal. However, FLGC also sacrifices the accuracy a lot in order to reach such a reduction on computational cost. On the contrary, our proposed LdsConv mainly transforms the 3×3 convolution into the depthwise separable one and make a sweet balance between the FLOPs and the accuracy. ND decomposes the regular convolution into the accumulation of several depthwise separable convolutions. While our approach aims to replace the standard convolution with a single depthwise separable convolution. Further more, our Lds-ResNet50-extreme replaces with only one depthwise convolution (w/o separable one) resulting a extreme reduction on computation cost which can be never transcended by ND. The goal of DGConv is to construct a groupwise convolution with dynamic groups. While our approach is to construct a depthwise (Lds-ResNet50-extreme) or depthwise separable convolution with most valuable filters. Our Lds-ResNet50-extreme plays a role as the upper bound of reduction on FLOPs for DGConv-ResNet50 and our Lds-ResNet50${}^{*}$ simply surpasses the accuracy with fewer extra FLOPs. As shown in Table 5, our Lds-ResNet50${}^{*}$ outperforms other methods in terms of accuracy and still has a considerable reduction on FLOPs and number of parameters. Our Lds-ResNet50-extreme remains a comparable accuracy with strong compression on the model.

### 4.4. Network Visualization with Grad-CAM

**Figure 7:**
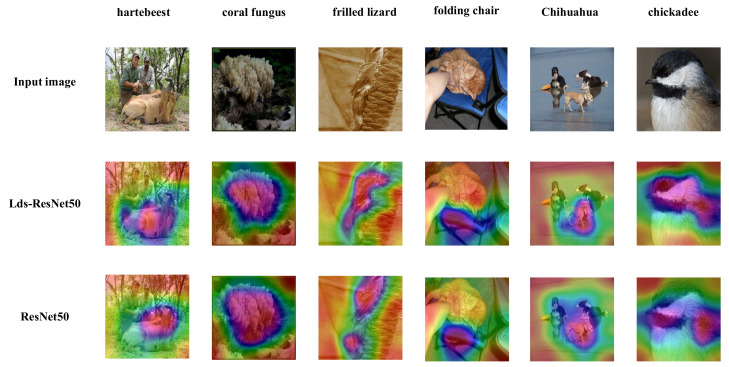
Grad-CAM [58] visualization results. We compare the visualization results between our Lds-ResNet50 and ResNet50. The Grad-CAM visualization is calculated for the last convolutional outputs. The ground-truth label is shown on the top of each input image.

We further apply the Grad-CAM [58] to models using images from the ImageNet validation set. Grad-CAM uses gradients to calculate the importance of the spatial locations in convolutional layers. As the gradients are calculate with respect to a specific class, Grad-CAM results show attended regions clearly. By visualizing the importance map for the network, we are able to understand which part the network is interested in and how the network is making use of the features for predicting a class. We compare the visualization results between our proposed Lds-ResNet50 and baseline (ResNet50) in Figure 7.

From Figure 7 it can be clearly seen that the Grad-CAM results of Lds-ResNet50 cover the target regions better than those of the original ResNet50. It suggests that LdsConv-integrated network learns well to exploit information in target regions and aggregate features from them.

## 5. Conclusions

In this work, we propose a new type of convolution called LdsConv. We have compared our proposed convolutional filters with the original convolutional filters on various existing architectures. Experimental results show that our LdsConv is more efficient than existing convolutions in these models. We also have compared the LdsConv method with the FLOPs compression methods and similar motivated works. Results from our experiments show that the proposed method produces the overall best accuracy while still having competitive FLOPs.

## Figures and Tables

**Table 1:** The table shows the ablation study results in different setups on CIFAR-100. ‘${}^{*}$’ refers to the LdsConv using the balance loss. ‘${}^{\#}$’ refers to the model trained with gap epochs $E_{G}=E_{P}/4$.

Model	Accuracy (%)	GFLOPs	Params (M)
Lds-DenseNet-BC-100 (*s* = 2)	76.9	0.23	0.64
Lds-DenseNet-BC-100 (*s* = 4)	77.3	0.23	0.64
Lds-DenseNet-BC-100 (*s* = 6)	76.3	0.23	0.64
Lds-DenseNet-BC-100 (*s* = 8)	76.6	0.23	0.64
Lds-DenseNet-BC-100 ${}^{\#}$ (*s* = 6)	77.4	0.23	0.64
Lds-DenseNet-BC-100 ${}^{\#}$ (*s* = 8)	77.9	0.23	0.64
Lds-DenseNet-BC-100 (*k* = 1)	76.3	0.21	0.6
Lds-DenseNet-BC-100 (*k* = 2)	77.3	0.23	0.64
Lds-DenseNet-BC-100 (*k* = 3)	77.3	0.25	0.71
Lds-DenseNet-BC-100 (*k* = 4)	76.8	0.28	0.74
Lds-DenseNet-BC-100 ${}^{*}$ (*k* = 1)	76.8	0.21	0.6
Lds-DenseNet-BC-100 ${}^{*}$ (*k* = 2)	77.7	0.23	0.64
Lds-DenseNet-BC-100 ${}^{*}$ (*k* = 3)	77.6	0.25	0.71
Lds-DenseNet-BC-100 ${}^{*}$ (*k* = 4)	77.1	0.28	0.74
Lds-ResNet50 ${}^{*}$ (N${}_{O}$ = 4)	80.1	2.87	14.97
Lds-ResNet50 ${}^{*}$ (N${}_{O}$ = 8)	80.9	2.87	14.97
Lds-ResNet50 ${}^{*}$ (N${}_{O}$ = 16)	79.8	2.87	14.97
Lds-ResNet50 ${}^{*}$ (N${}_{O}$ = 32)	79.8	2.87	14.97
Dw-DenseNet-BC-100	74.6	0.21	0.6
Lds-DenseNet-BC-100 (*k* = 2) w/o AC	76.2	0.3	0.79

**Table 2:** The table shows the results for different models on CIFAR-100. ‘${}^{*}$’ refers to the LdsConv using the balance loss. With the setting obtained from the ablation study, we can simply improve the performance of the model by replacing the standard 3 × 3 convolution with our proposed LdsConv.

Model	Accuracy (%)	GFLOPs	Params (M)
MobileNet [9]	77.1	0.62	3.31
Lds-MobileNet ${}^{*}$	78.0	0.51	2.74
ResNet50 [1]	80.2	4.46	23.71
Lds-ResNet50 ${}^{*}$	80.9	2.86	14.97
ResNet152 [1]	81.7	14.20	58.34
Lds-ResNet152 ${}^{*}$	82.0	8.66	35.53
SE-ResNet50 [43]	81.2	4.46	26.22
Lds-SE-ResNet50 ${}^{*}$	81.5	2.87	16.54
DenseNet-BC-100 [42]	77.7	0.30	0.79
Lds-DenseNet-BC-100 ${}^{*}$	77.7	0.23	0.64

**Table 3:** The table shows the results for different models on ImageNet. ‘${}^{*}$’ refers to the LdsConv using the balance loss. By simply replacing the standard 3 × 3 convolutional filters with our proposed LdsConv filters, we can not only improve the accuracy but also reduce the FLOPs and the number of parameters a lot. For the case of MobileNet, we highly increase the accuracy by 2.3% which is a pretty considerable improvement.

Model	Error% (Top-1)	GFLOPs	Params (M)
MobileNet [9]	29.0	0.57	4.2
Lds-MobileNet ${}^{*}$	26.7	0.49	3.7
ResNet50 [1]	24.7	3.86	25.6
Lds-ResNet50 ${}^{*}$	22.9	2.71	16.8
ResNet152 [1]	23.0	11.30	60.2
Lds-ResNet152 ${}^{*}$	21.2	7.14	37.4
SE-ResNet50 [43]	23.3	3.87	28.1
Lds-SE-ResNet50 ${}^{*}$	22.0	2.71	17.1
SE-ResNet152 [43]	21.6	11.32	66.8
Lds-SE-ResNet152 ${}^{*}$	20.7	7.15	38.2
DenseNet121 [42]	25.0	2.88	8.0
Lds-DenseNet121 ${}^{*}$	24.2	1.99	6.5
DenseNet264 [42]	22.2	5.86	33.3
Lds-DenseNet264 ${}^{*}$	21.7	4.72	29.9

**Table 4:** The table shows the comparison with existing compression methods for ResNet50 on ImageNet. Our Lds-ResNet50-extreme outperforms all other methods in terms of accuracy and still has a comparable reduction on FLOPs.

Model	Error% (Top-1)	GFLOPs	FLOPs↓ (%)
ThiNet-70 [30]	27.9	-	36.8
NISP [56]	27.3	-	27.3
FPGM-only 30% [57]	24.4	-	42.2
Lds-ResNet50-extreme	23.4	2.28	40.9

**Table 5:** The table shows the comparison with similar methods for ResNet50 on ImageNet. ‘${}^{*}$’ refers to the LdsConv using the balance loss.

Model	Error% (Top-1)	GFLOPs	Params (M)
FLGC-ResNet50 [48]	34.2	1.0	7.6
ND-ResNet50 [50]	26.7	3.12	20.6
DGConv-ResNet50 [49]	23.3	2.46	14.6
Lds-ResNet50 ${}^{*}$	22.9	2.71	16.8
Lds-ResNet50-extreme	23.4	2.28	14.3
